# The effect of *Coriandrum sativum* L. on yogurt: a comprehensive study of physicochemical, microbiological, sensory, and textural properties

**DOI:** 10.3389/fnut.2024.1493602

**Published:** 2025-01-06

**Authors:** Tamer Turgut, Abdulkerim Diler

**Affiliations:** ^1^Department of Food Processing, Vocational College of Technical Sciences, Atatürk University, Erzurum, Türkiye; ^2^Department of Plant and Animal Production, Vocational College of Technical Sciences, Atatürk University, Erzurum, Türkiye

**Keywords:** aromatic plants, color parameters, *Coriandrum sativum* L., functional yogurt, microbiological viability

## Abstract

**Objectives:**

The objective of this study was to investigate the effects of the addition of *Coriandrum sativum* L. (coriander) on the physicochemical, sensory, textural and microbiological properties of yogurt.

**Methods:**

To conduct this study, 4 types of yogurt were prepared as control (C0) and with 1% (C1), 2% (C2) and 3% (C3) coriander, and the yogurts were analyzed on specific storage days.

**Results:**

The addition of coriander significantly influenced some physiochemical parameters (syneresis, titratable acidity) of yogurts (*p* < 0.05). The storage time significantly affected the water holding capacity (WHC), syneresis and titratable acidity (TA). The TA of the yogurts increased during storage. The addition of coriander significantly influenced the color parameters (ΔE, C* and YI) of yogurts (*p* < 0.05). The results of the statistical analysis indicated that storage time had a significant effect on the viability of yogurt bacteria (*p* < 0.05). While the number of *Lactobacillus bulgaricus* decreased, an increase was observed in the number of *Streptococcus thermophilus*. The number of both yogurt bacteria remained above 7.46 log cfu/g throughout storage. The addition of coriander to yogurt led to changes in the number of yogurt bacteria. When the texture parameters of the yogurts were compared, no significant difference was seen between the yogurts. The sensory evaluation showed that yogurts with coriander had a higher overall acceptability than C0 yogurts.

**Conclusion:**

The results of the research indicate that the addition of coriander has a positive effect on the quality of yogurt. The results of this study suggest that the inclusion of coriander and other similar aromatic plants in yogurt production could be beneficial in terms of producing functional yogurt and the potential uses of these plants should be investigated.

## 1 Introduction

Yogurts have always had a very important place in studies examining the relationship between nutrition and human health. Most foods on the market are dairy-based and the health benefits of dairy products containing live bacteria have been proven. Yogurt is a fresh fermented milk product made by fermenting milk with *Streptococcus thermophilus* and *Lactobacillus delbrueckii* subsp. *bulgaricus* (1–3). Possible health benefits of regular yogurt consumption include lowering cholesterol levels, digestive support, reducing weight gain, reducing gastrointestinal infections and risk of colon cancer, as well as preventing obesity, strengthening the immune system, preventing diabetes, promoting calcium absorption, and eliminating symptoms of lactose intolerance (4, 5). Coriander is also attracting attention for its antimicrobial, antioxidant and protective properties (6).

Lactic acid bacteria are famous for their antimicrobial, antiviral, and immunomodulatory properties and are involved in the treatment of many gastrointestinal diseases (7). Whether yogurt bacteria should be regarded as probiotics is being debated by various scientists. *S. thermophilus* and *L. bulgaricus* were not considered probiotic bacteria by some scientists because they are not tolerant of acidic environments in the stomach and do not adhere to the intestinal wall and multiply (8). However, current scientific consensus is that yogurt cultures must be considered probiotics if some of the health benefits obtained from yogurt consumption are linked to the presence of live bacteria. (Probiotics had been defined as “live food supplements beneficial to health”) (5, 9, 10). Yogurt cultures can also be probiotic, provided they meet this definition. It has been confirmed by the European Food Safety Authority that *S. thermophilus* and *L. bulgaricus* improve lactose digestion (5). Another example of the beneficial effect of yogurt bacteria is improving vitamin B levels in adults (10). Thus, yogurt starter cultures fulfill the current concept of probiotics, at least as far as their positive effect on lactose digestion *in vivo* is concerned.

Aromatic plants play a very crucial role in the prevention of chronic diseases such as heart disease, cancer, diabetes and Alzheimer’s (11). Recent studies have shown that these plants contain bioactive compounds, so-called phytochemicals, which reduce oxidative stress and regulate harmful biological processes. They are also said to have therapeutic properties (43). The use of botanicals with medicinal properties in yogurt has been studied by many researchers (12–14).

*Coriandrum sativum* L. (coriander or cilantro) is an aromatic plant and belongs to the parsley (Apiaceae) family. Although its homeland is the Mediterranean region, it also grows in Asia, the Middle East, North Africa region, and Central Europe (7, 15). Coriander is often used for its fresh leaves and seeds, which have organoleptic and flavor properties. Due to its nutritional and medicinal properties, it has been one of the most widely used medicinal plants since ancient times (6). Coriander and its essential oil has a broad spectrum of biological activities, such as antimicrobial, antioxidant, antidiabetic, antiepileptic, antimutagenic, anti-inflammatory, antihypertensive, and diuretic effects (6, 16).

The objectives of this study are (i) to determine how the addition of coriander at different concentrations affects various yogurt properties such as physicochemical, microbiological and texture, and (ii) to understand how the addition of coriander affects consumer acceptance. Furthermore, the study suggests that adding coriander to yogurt, which has many important benefits including antibacterial, antioxidant and anti-inflammatory effects, could help improve public health.

## 2 Materials and methods

### 2.1 Materials

Cattle raw milk was purchased from the Ataturk University Livestock Practice Center (Erzurum, Turkey). The mean number of somatic cells (SSC) in the milk was 95.000 per ml of milk. DVS YC-381 YoFlex® yogurt culture (*L. bulgaricus* and *S. thermophilus*) was donated by Chr Hansen (Istanbul, Turkey) and used as described by the manufacturer. Fresh coriander plants were purchased from a local supermarket (Erzurum). The leaves of the plant were removed from their stems, the extracted leaves were washed with plenty of water, then finely chopped and heated at 82°C for 6 min to reduce its microbial load. After cooling, the coriander leaf was thoroughly filtered, placed in a glass jar, and stored in the refrigerator until it was used in the yogurts.

### 2.2 Production of yogurt

Raw milk was evaporated to achieve 12% skim milk solids under vacuum pressure (450 mmHg) at 60°C and heated to 90°C for 5 min then cooled down to 44 ± 1°C specific temperature. The primary culture was prepared by dissolving one-quarter of a 50-unit bag in 100 mL of milk. The primary culture (17 mL) was mixed with 10 L of milk and divided into four equal batches. The batches were incubated at a controlled temperature of 44 ± 1°C until pH 4.5 was reached. The pH is a measure of acidity and an important indicator of when fermentation is complete in yogurt production. After the incubation process, the yogurt batches were placed in a fridge to cool and harden and then stored at 4°C overnight. One yogurt group was separated as the control yogurt (C0) and coriander leaves (cilantro) were added to the remaining yogurt groups in different percentages: 1.0% (C1), 2% (C2), and 3% (C3) (w/w). After the coriander was added, the stirred yogurts were filled into 150 mL sterile bottles. This temperature (4°C) is common for refrigerated storage and is suitable for preserving the yogurt. Analyzes were performed twice on the 2nd, 7th, 14th, and 21st day of storage. In this study, yogurt production was performed in two replicates and the analysis was carried out in two parallel experiments.

### 2.3 Microbiological analyses

Ten grams of each yogurt sample was weighed and a 10:1 dilution was prepared by shaking with 90 mL of Ringer’s diluent for 1 min. Subsequent dilutions were continued from the 10^−1^ dilution with Ringer’s dilution. The dilution process was continued until a 10^−7^ dilution was achieved, after which the appropriate dilutions were spread on petri plates according to the spreading technique. The enumeration of *S. thermophilus* was conducted on M17 agar (Merck, Germany) at 37°C for 48 h. The enumeration of *L. bulgaricus* was performed on MRS agar (Merck) and incubated anaerobically at 37°C for 48–72 h (17). Total viable count (TVC) was enumerated on PCA agar (Merck) at 30–32°C for 48 h. Coliform bacteria were enumerated on VRB agar (Merck) and incubated aerobically at 35–37°C for 24 h (18). Plates containing 30–300 colonies were evaluated and the results were given in the form of CFUg^−1^.

### 2.4 Physicochemical analyses

The dry matter of yogurt samples was calculated using the drying them in an oven method. The fat content of the yogurt samples was determined using the Gerber method based on the AOAC (19) method. The pH measurements were performed regularly with a pH meter (Hanna, Romania) at room temperature. The titratable acidity (TA) was calculated according to the titration method (44). The syneresis refers to the separation of the whey phase from the yogurt. While the WHC refers to its retention. Both properties were measured using the method described by Turgut and Diler (7). For syneresis, 25 g of yogurt samples were weighed and filtered for 2 h at 4°C via a funnel using filter paper (Whatman No. 1, UK). Equation 1 was used to determine syneresis. For WHC, 10 g of yogurt samples were centrifuged (4500 × g for 30 min at 4°C). The filtrates were weighed, and the WHC values were calculated according to Equation 2.


(1) $$\mathrm{Syneresis}\;\left(\%\right)=\frac{\mathrm{whey}\;\mathrm{volume}}{\mathrm{initial}\;\mathrm{volume}}\times 100$$

(2) $$WHC\;\left(\%\right)=\left[1-\frac{\mathrm{filtrate}\;\mathrm{weight}}{\mathrm{initial}\;\mathrm{weight}}\right]\times 100$$

The color measurement was performed with a PCE XXM-20 colourimeter (PCE Instrument) with illuminant LED. The color evaluation of the yogurt samples was determined by coordinating the measured values according to the CIE-Lab scale, where L* indicates brightness, a* and b* indicate chromaticity coordinates. Each yogurt sample was evaluated at least 6 randomly selected positions. The yogurts were kept between 10 and 12°C during the analyses. The color difference (∆E) was calculated using Equation 3. This analysis is used to evaluate between the color of yogurts and the model of ideal whiteness. The chroma (C*) and yellowing index (YI) were calculated using Equations 4, 5.


(3) $$\left(\Delta E\right)=\sqrt{\left(\Delta L^{*}\right)+{\left(\Delta a^{*}\right)}^{2}+{\left(\Delta b^{*}\right)}^{2}}$$

(4) $$C^{*}=\sqrt{{\left(\Delta a^{*}\right)}^{2}+{\left(\Delta b^{*}\right)}^{2}}$$

(5) $$YI=142.86.b^{*}.L^{-1}$$

L* stands for lightness, a* for the redness b* for the yellowing and Δa = a_i_ − a_0_, Δb = b_i_ − b_0_ and ΔL = L_i_ − L_0_. The i stands for the observed value for each storage time (2, 7, 14, and 21) and index 0 for the references used. In the study, we have the colur parameters of the white standard (L: 100, a: 0, b:0).

### 2.5 Sensory analyses

The sensory evaluation of the yogurt samples was conducted by eight trained experienced panelists. The panelists rated various sensory properties (odor, syneresis, texture, acidity, flavor, sweetness and acceptability) of the yogurts on a scale from 1 to 9 according to the method outlined by Roland et al. (20). This 9-point rated scale model allowed a quantitative evaluation of sensory properties. All yogurt samples were given to participants in a bottle (150 mL) at 5°C.

### 2.6 Texture analyses

Texture analyses (TPA) of yogurts were carried out by penetration test using a TA XT2 Texture Analyser (Stable Micro Systems, UK) combined with an extruded P25 probe. The probe penetrated from the top of the yogurt samples to a depth of 30 mm at a speed of 1 mm s^−1^. Before the test, the compression and expansion speeds of the instrument were programmed to 1.0 mm s^−1^ (The specific probe and depth provide standardized conditions for testing the samples) (21). The following parameters were measured: adhesiveness, hardness, cohesiveness, springiness, gumminess, chewiness, and resilience. The breaking force corresponds to the first peak, the hardness corresponds to the maximum force and adhesion corresponds to the resistance with which the probe is pulled through the sample. The areal distance curve was calculated with the TPA exponent software package program. The values were analyzed with the Exponent software (Version 4.0.90). The software calculates the data with many formulas and gives texture analysis results.

### 2.7 Statistical analysis

Statistical analyses were performed using SPSS 20.0 on the Windows 10 OS. A generalized linear statistical model was employed for the analysis. Various physiochemical, microbiological, and textural analysis results obtained during the storage period of the study were analysed. Statistical tests were evaluated at the significance level of *α* = 0.05 and *α* = 0.01. Where means were significant, the Duncan Multiple Range test was used to compare yogurt samples.

## 3 Results and discussion

### 3.1 Measurement of acidity (pH and TA)

Table 1 summarizes some physicochemical analysis results of the yogurt samples. The effect of storage time on the pH and TA values of the yogurt samples was significant (*p* < 0.01). The mean TA value of the yogurts was between 1.16% (C0) and 1.24% (C2). The lowest TA value was 1.09% was on day 2 and the highest 1.30% on day 21. The mean pH value, which was 4.28 at the beginning of storage, dropped to 3.99 on the 21st day. However, after the 14th day, the differences between the mean values were no longer statistically significant (*p* > 0.05). These results clearly show that the acidity of the yogurt samples increased gradually during storage. The increase in acidity is mainly due to the survival and activity of the cultures used in yogurt production. The reason for this is the post-acidification process, whereby the yogurt bacteria become active during storage. Bakırcı and Kavaz (22) also came to similar conclusions. Çakmakçı et al. (23) found that the pH of yogurt samples containing probiotics exhibited a range of values between 4.07 and 4.60. The low pH value in their study could have been caused by the probiotic bacteria used by the researchers.

**Table 1:** The changes in physicochemical characteristics of yogurt samples during storage.

	Days	Total Solids (%) $\bar{x}\pm SD$	Fat (%) $\bar{x}\pm SD$	Syneresis (%) $\bar{x}\pm SD$	WHC (%) $\bar{x}\pm SD$	Tit. acidity (%) $\bar{x}\pm SD$	pH $\bar{x}\pm SD$
Yogurt samples							
C0	2	13.93	3.51	40.47	46.92	1.04	4.29
	7	14.37	3.74	39.00	52.44	1.15	4.16
	14	14.19	3.83	37.60	47.74	1.20	4.04
	21	14.55	3.77	36.66	52.41	1.25	4.01
	Mean	14.26 ± 0.599	3.96 ± 0.260^bc^	38.43 ± 1.835^b^	49.88 ± 3.026	1.16 ± 0.045^a^	4.13 ± 0.105
C1	2	13.38	3.63	39.03	46.69	1.09	4.25
	7	13.81	3.87	37.56	52.20	1.20	4.12
	14	13.64	3.96	36.16	47.50	1.25	4.00
	21	14.00	3.89	35.22	52.17	1.31	3.97
	Mean	13.71 ± 0.596	4.08 ± 0.254^c^	36.99 ± 1.827^ab^	49.64 ± 3.032	1.21 ± 0.042^b^	4.09 ± 0.111
C2	2	13.78	3.44	37.58	46.57	1.12	4.29
	7	14.21	3.68	36.11	52.09	1.23	4.15
	14	14.04	3.77	34.71	47.39	1.28	4.04
	21	14.40	3.71	33.77	52.06	1.33	4.01
	Mean	14.11 ± 0.605	3.81 ± 0.261^ab^	35.50 ± 1.832^a^	49.53 ± 3.029	1.24 ± 0.051^b^	4.12 ± 0.108
C3	2	13.72	3.52	37.58	46.12	1.10	4.27
	7	14.15	3.76	36.11	52.44	1.22	4.13
	14	13.98	3.84	34.71	52.20	1.27	4.01
	21	14.33	3.78	33.78	52.09	1.32	3.98
	Mean	14.04 ± 0.601	3.66 ± 0.257^a^	35.55 ± 1.830^a^	49.08 ± 3.034	1.23 ± 0.048^b^	4.10 ± 0.110

Storage time (Mean)													
Days	*N*												
2	8	13.71 ± 0.602		3.75 ± 0.263		38.67 ± 1.840^c^		46.58 ± 3.027^a^		1.09 ± 0.043^a^		4.28 ± 0.099^c^	
7	8	14.14 ± 0.604		3.93 ± 0.260		37.19 ± 1.830^bc^		52.09 ± 3.033^b^		1.20 ± 0.047^b^		4.14 ± 0.096^b^	
14	8	13.97 ± 0.596		3.91 ± 0.254		35.79 ± 1.829^ab^		47.39 ± 3.030^a^		1.25 ± 0.044^b^		4.02 ± 0.107^a^	
21	8	14.32 ± 0.591		3.93 ± 0.265		34.86 ± 1.832^a^		52.06 ± 3.035^b^		1.30 ± 0.039^c^		3.99 ± 0.110^a^	
Source	D.F												
Yogurt samples	3		NS		**		**		NS		**		NS
Storage time	3		NS		NS		**		**		**		**
Error	24												
Total	31												

C0, control yogurt (0%); C1, yogurt with 1% coriander; C2, yogurt with 2% coriander; C3, yogurt with 3% coriander. ^a,b,c^Means in the same column without a common superscript differ (*p* < 0.05). **are significant at 0.01 probability levels. NS, not significant.

The addition of coriander significantly affected the TA values of the yogurt samples (*p* < 0.01). A lower level of TA was found in C0 yogurt (1.16%) than in yogurts with coriander added. Aydemir and Altun (24) confirm the results of our study by reporting during storage of yogurts, syneresis and pH decreased, while lactic acid increased. However, the incorporation of coriander did not markedly alter the pH values of the yogurt samples. The pH values of the yogurts with coriander (C1, C2, and C3) were slightly lower than those of the control yogurt (C0), and differences between the means were insignificant (*p* > 0.05). This indicates that coriander does not cause any significant change in pH values. Gündogdu et al. (45) stated no significant differences in acidity levels between control and garlic-enriched yogurts (0.5 and 1%), reinforcing the notion that live starter bacteria primarily drive acidity increase over time. The results show that the primary factor affecting the acidity increase in the trial yogurts is storage time. However, the addition of coriander was also found to have a remarkable effect on TA.

### 3.2 Syneresis and WHC

The syneresis and WHC values of the yogurt samples are summarized in Table 1. Coriander addition was found to be significant for syneresis (*p* < 0.05), but insignificant for WHC (*p* > 0.05). Storage time caused a significant difference in both properties (*p* < 0.05). It was observed that the syneresis values of yogurts decreased in a proportional manner with the addition of coriander. The highest syneresis values were found in C0 yogurt (38.43%), while the lowest values were found in C2 yogurt (35.50%). The observed reduction can be explained by the binding of phenolic compounds from coriander to milk proteins (25). One of the principal causes of syneresis in yogurt samples is a drop in pH, which is a prevalent issue. However, in the presented study, no significant difference in pH values was observed. This significant difference in syneresis is due to the proportionality of the weight of coriander added. The syneresis values decreased during storage, possibly due to the increased dry matter content over time or the presence of coriander. Other studies, such as the one by Korkmaz et al. (26) also observed a decrease in syneresis values of yogurt enriched with propolis and *Lepidium meyenii* powder. The highest WHC value (52.09) observed on day 7. The addition of coriander resulted in a slight decrease in WHC values. The WHC values of the yogurts with coriander were lower than those of the C0 yogurt, but differences between the means were insignificant (*p* > 0.05). Nevertheless, the WHC values observed in this study were found to be lower than those previously reported by Karaca et al. (27).

### 3.3 Viable counts

In addition to providing the host with an extremely high number of viable bacteria, yogurt can also provide other benefits to human health (28). The microbiological evaluation results for *S. thermophilus*, *L. bulgaricus*, TVC, and coliform bacteria determined in all yogurts are summarized in Table 2. Coliform counts were below detectable limits (2 log cfu/g) in all yogurt samples, indicating good microbiological quality. The results show that both coriander addition and storage time significantly affected the number of *S. thermophilus* in the yogurts (*p* < 0.05). The number of *S. thermophilus* increased during storage and peaked on day 14 in the C0 yogurt. This increase in the number of *S. thermophilus* correlated with the increase in TA of the yogurts during storage, suggesting that the proliferation of *S. thermophilus* contributed to the increase in acidity. This is due to the fact that the number of the second of the two bacteria (*L. bulgaricus*) decreased during this period. The storage time significantly influenced the survival rate of *L. bulgaricus* in yogurt samples. The results show that a longer storage time is a greater source of stress. Beal et al. (29) stated that *S. thermophilus* can adapt to unfavorable growth conditions corresponding to low pH. This observation is in accordance with the results of our study. The addition of coriander was found to reduce the number of *S. thermophilus* and *L. bulgaricus* in the yogurt samples. This reduction could be attributed to the presence of some compounds in coriander that could inhibit the growth of the yogurt starter bacteria. The essential oil of coriander has been demonstrated to be effective against a variety of foodborne bacteria (16). The addition of coriander to the yogurt samples resulted in a notable reduction in the number of *S. thermophilus* (0.42 log cfu/g) (*p* < 0.05), although these reductions were not significant for the number of *L. bulgaricus* (*p* > 0.05). The presence of coriander in yogurt may slow down the overgrowth of yogurt cultures required for fermentation of yogurt, thereby extending the shelf life of yogurt. The results match previous studies that showed a reduction in the number of *S. thermophilus* and *L. bulgaricus* during the storage period. Some studies indicated that *L. bulgaricus* exhibited a faster decline than *S. thermophilus* (30). In a separate study, the number of *L. bulgaricus* increased by approximately 0.4 log units during storage (24). Birollo et al. (31) observed that *S. thermophilus* and *L. bulgaricus* increased slightly in yogurts over a six-week period and then decreased. Akın and Akın (32) found that the number of both bacteria decreased by about one log unit during 14 days of storage. In this study, the numbers of *S. thermophilus* increased by 0.35 log units and the numbers of *L. bulgaricus* decreased by 0.42 log units during the 21 days of storage. It is therefore important to understand these differences and to optimize the conditions to maintain the viability of the health-promoting yogurt bacteria.

**Table 2:** The changes in microbiological characteristics of yogurt samples during storage.

	N	*L. bulgaricus* (log cfu/g) $\bar{x}\pm SD$	*S. thermophilus* (log cfu/g) $\bar{x}\pm SD$	TVC (log cfu/g) $\bar{x}\pm SD$	Coliform *(*log cfu/g)
Yogurt samples					
C0	8	7.96 ± 0.597	8.44 ± 0.331^b^	8.07 ± 0.333^a^	<2
C1	8	7.76 ± 0.603	8.27 ± 0.355^ab^	8.06 ± 0.329^a^	<2
C2	8	7.85 ± 0.599	8.15 ± 0.319^ab^	8.46 ± 0.340^b^	<2
C3	8	7.71 ± 0.589	8.02 ± 0.328^a^	8.49 ± 0.336^b^	<2
Storage time (days)					
2	8	7.89 ± 0.608^ab^	7.93 ± 0.319^a^	8.22 ± 0.342	<2
7	8	8.31 ± 0.590^b^	8.31 ± 0.330^b^	8.22 ± 0.330	<2
14	8	7.63 ± 0.605^a^	8.35 ± 0.325^b^	8.16 ± 0.339	<2
21	8	7.46 ± 0.602^a^	8.28 ± 0.334^b^	8.48 ± 0.334	<2
Source	D.F				
Yogurt samples	3	NS	*	*	NS
Storage time	3	*	*	NS	NS
Error	24				
Total	31				

C0, control yogurt (0%); C1, yogurt with 1% coriander; C2, yogurt with 2% coriander; C3, yogurt with 3% coriander. ^a,b^means in the same column without a common superscript differ (p < 0.05).

The incorporation of coriander significantly influenced the TVC in the yogurt samples (*p* < 0.05). The mean TVC varied among the yogurts, with the highest TVC observed in the C3 yogurts and the lowest in the C1 yogurts (Table 2). While there were slight increases in TVC during storage, these increases were not statistically significant (*p* > 0.05). The initial mean TVC value of yogurts was 8.22 log cfu/g, and this value increased to 8.48 log cfu/g on the 21st day of storage. Turgut and Diler (7) reported an increase of only 0.02 log units within 21 days in yogurts containing loquat. The results contribute to our understanding of the potential effects of self-life and storage conditions of yogurt, as well as complementary ingredients such as coriander, on bacterial growth.

### 3.4 Color analyses

The color and taste of food are two components that complement each other. Color, in particular, is a key criterion that influences consumer preferences at first glance. The color of yogurt is a significant indicator that influences the quality, freshness, taste expectation and acceptance of the product. Yogurt has a bright white color, which is due to the milk fat, proteins and natural pigments in its composition (33). Table 3 provides an overview of the changes in the color parameter of the yogurts. The addition of coriander had a significant effect (*p* < 0.01) on the color parameters of the yogurts, with a higher proportion of coriander making the color appear more greenish-yellow. As the amount of added coriander increased, the L* and a* parameters exhibited a gradual decline, while b* value exhibited a gradual increase. Lower L* and a* parameters indicate less white and green content in the yogurts, while higher b* values indicate a predominance of yellow. The highest numerical L*, a* and b* values were measured for the C0 yogurt (Table 3). The L* and b* values of the C0 yogurt and the yogurts with coriander showed a slight increase during the storage period. No significant differences were found in the L* value during the storage period. The storage time only significantly influenced the a* value of the yogurts (*p* < 0.05). The decrease in b* values during storage was insignificant. The yogurts with coriander became greener during storage, as shown by the change in the C* value.

**Table 3:** The color properties of the yogurt samples during storage.

	Days	L* $\bar{x}\pm SD$	a* $\bar{x}\pm SD$	b* $\bar{x}\pm SD$	∆E $\bar{x}\pm SD$	C* $\bar{x}\pm SD$	YI $\bar{x}\pm SD$
Yogurt samples							
C0	2	86.50	−45.37	8.29	48.06	46.12	13.68
	7	86.08	−45.90	8.09	48.64	46.61	13.43
	14	85.47	−43.81	8.51	46.94	44.63	14.22
	21	85.96	−42.21	8.45	45.27	43.04	14.04
	Mean	86.00 ± 0.73^c^	−44.32 ± 2.06^a^	8.33 ± 0.74^c^	47.22 ± 1.89^a^	45.10 ± 1.87^a^	13.84 ± 1.28^c^
C1	2	84.37	−48.19	6.36	51.05	48.60	10.76
	7	83.95	−48.71	6.17	51.66	49.10	10.49
	14	83.34	−46.63	6.58	49.95	47.09	11.28
	21	83.83	−45.02	6.52	48.28	45.49	11.11
	Mean	83.87 ± 0.76^b^	−47.14 ± 2.01^b^	6.41 ± 0.65^ab^	50.23 ± 2.02^b^	47.57 ± 1.83^b^	10.92 ± 1.21^b^
C2	2	83.98	−48.68	6.04	51.60	49.05	10.27
	7	83.57	−48.30	6.01	51.37	48.68	10.27
	14	82.96	−47.72	6.24	51.05	48.12	10.75
	21	83.44	−45.81	6.22	49.10	46.23	10.65
	Mean	83.49 ± 0.79^b^	−47.63 ± 1.98^bc^	6.13 ± 0.71^b^	50.78 ± 1.95^b^	48.02 ± 1.86^bc^	10.49 ± 1.19^ab^
C3	2	83.00	−48.86	5.85	52.07	49.21	10.06
	7	82.58	−49.39	5.66	52.68	49.71	9.78
	14	81.97	−47.30	6.07	50.98	47.69	10.58
	21	82.46	−45.69	6.01	49.31	46.09	10.41
	Mean	82.50 ± 0.70^a^	−47.81 ± 2.04^c^	5.90 ± 0.76^a^	51.25 ± 1.98^c^	48.17 ± 1.81^c^	10.22 ± 1.22^a^

Storage time (Mean)							
Days	*N*						
2	8	84.46 ± 0.80	−47.27 ± 2.02^c^	6.89 ± 0.72	50.23 ± 1.94^b^	47.77 ± 1.95^b^	11.65 ± 1.24
7	8	84.05 ± 0.75	−47.80 ± 1.95^c^	6.70 ± 0.67	50.83 ± 1.94^b^	48.27 ± 1.87^b^	11.39 ± 1.27
14	8	83.44 ± 0.76	−45.71 ± 2.03^ab^	7.12 ± 0.73	49.14 ± 1.97^ab^	46.26 ± 1.87^ab^	12.19 ± 1.25
21	8	83.92 ± 0.82	−44.11 ± 1.97^a^	7.05 ± 0.64	47.48 ± 1.88^a^	44.67 ± 1.85^a^	12.01 ± 1.16
Source	D.F						
Yogurt samples	3	**	**	**	**	**	**
Storage time	3	NS	**	NS	**	**	NS
Error	24						
Total	31						

C0, control yogurt (0%); C1, yogurt with 1% coriander; C2, yogurt with 2% coriander; C3, yogurt with 3% coriander. L*: lightness, scale 0 (black) to 100 (white); a*: redness, + a* (red) to – a* (green); b*: yellowness, + b* (yellow) to – b* (blue); ∆E: color difference; C*: chroma; YI: yellowing index. ^a,b,c^means in the same column without a common superscript differ (*p* < 0.05). **are significant at 0.01 probability levels. NS, not significant.

Park et al. (34) reported that temperature or Maillard reaction was responsible for the increase in b* value during storage. Since the yogurt in the present study was stored under refrigerated conditions, it is normal that the b* value does not increase. This indicates that the specific pigments contained in coriander withstand the conditions of yogurt production and storage, so that the color of the yogurt remains relatively constant over time. Ścibisz et al. (35) stated that the color changes caused by the addition of blueberries depended on both the amount of fruit and storage. The color parameters ΔE, which represents the total color difference between two samples, Chroma (C^*^), also known as saturation, which represents the intensity or strength of a color, and Yellowness Index (YI), a specific measure of the yellowness or greenness of a sample, were significantly changed by the addition of coriander (*p* < 0.01). The values for color difference (ΔE) of yogurts ranged from 47.22 and 51.25 (Table 3). These results show a significant coloration in all yogurts analyzed. This was relatively consistent with the increase in the amount of coriander. This result can be said to be related to the sourness of yogurt. The higher the acidity, the less the coloration reaction (36). The highest yellowness index (YI) was found in C0 yogurt (Table 3). The YI of C2 yogurt was statistically similar to C1 and C3 yogurts. The ΔE and C* parameters changed significantly (p < 0.01) with the storage time. The ΔE values of yogurts decreased during storage.

### 3.5 Texture evaluation and sensory analyses

Texture evaluation of yogurts is important indicators of yogurt quality and plays a significant role in consumer perception and satisfaction (2, 30). The TPA data of yogurt samples was summarized in Table 4. The storage time significantly (*p* < 0.05) influenced the adhesiveness and resilience of the yogurts. Adhesiveness is important parameters for the evaluation of yogurt texture. Adhesiveness is a measure of yogurt stickiness. The adhesiveness and resilience values of the yogurts showed an increase during storage, indicating that the texture of yogurt changed over time. During storage, the adhesiveness value of the yogurts varied between −7.25 and −21.45, and the resilience value varied between 0.21 and 0.27. The adhesiveness and resilience values had significant (*p* < 0.05) difference between the 2nd and 21st day of storage. The TPA values of yogurts were not statistically affected by the addition of coriander. Similar results were reported in the studies of Espírito Santo et al. (21) and Delikanlı and Özcan (37). The addition of coriander had no significant effect on the TPA value of the yogurts (*p* > 0.05). However, yogurts with coriander exhibited a smoother texture than the C0 yogurt. The addition of coriander slightly decreased the hardness and increased the resilience and adhesiveness values, but these changes were not statistically significant (*p* > 0.05). Similar results were reported by Najgebauer-Lejko et al. (38) when yogurts were prepared with different vegetables.

**Table 4:** The textural properties of the yogurt samples during storage.

Days		Hardness (N) $\bar{x}\pm SD$	Adhesiveness (N.s) $\bar{x}\pm SD$	Springiness (−) $\bar{x}\pm SD$	Cohesiveness (−) $\bar{x}\pm SD$	Gumminess (N) $\bar{x}\pm SD$	Chewiness (N) $\bar{x}\pm SD$	Resilience (−) $\bar{x}\pm SD$
Yogurt samples								
C0	2	16.18	−8.65	1.00	0.80	12.85	12.80	0.26
	7	15.99	−15.05	0.99	0.78	12.50	12.35	0.23
	14	15.89	−18.52	0.98	0.79	12.01	11.48	0.24
	21	15.78	−22.86	0.97	0.78	12.68	12.26	0.20
Mean		15.97 ± 1.41	−16.26 ± 8.74	0.98 ± 0.03	0.79 ± 0.08	12.51 ± 0.87	12.22 ± 1.14	0.23 ± 0.02
C1	2	16.08	−6.86	0.99	0.80	12.65	12.49	0.27
	7	15.76	−13.23	0.98	0.78	12.29	12.04	0.24
	14	15.56	−17.70	0.97	0.79	11.99	11.17	0.25
	21	15.51	−21.05	0.96	0.78	12.49	11.95	0.21
Mean		15.73 ± 1.47	−14.71 ± 8.70	0.97 ± 0.06	0.79 ± 0.02	12.36 ± 093	11.91 ± 1.18	0.24 ± 0.05
C2	2	15.39	−6.57	0.99	0.82	12.64	12.55	0.27
	7	15.33	−12.94	0.98	0.80	12.28	12.11	0.24
	14	15.28	−17.41	0.97	0.81	11.96	11.23	0.25
	21	15.27	−20.76	0.96	0.80	12.47	12.02	0.21
Mean		15.32 ± 1.38	−14.17 ± 8.76	0.98 ± 0.02	0.81 ± 0.05	12.34 ± 0.96	11.98 ± 1.13	0.24 ± 0.06
C3	2	15.37	−6.91	0.99	0.81	12.38	12.26	0.27
	7	15.31	−13.28	0.98	0.79	12.02	11.81	0.24
	14	15.26	−17.75	0.97	0.80	11.71	10.94	0.25
	21	15.27	−21.11	0.96	0.78	12.22	11.72	0.21
Mean		15.30 ± 1.44	−14.76 ± 8.68	0.98 ± 0.05	0.79 ± 0.03	12.08 ± 0.91	11.68 ± 1.19	0.23 ± 0.02

**Storage time (Mean)**								
Days	*N*							
2	8	15.76 ± 1.27	−7.25 ± 8.03^b^	0.99 ± 0.06	0.81 ± 0.05	12.63 ± 0.94	12.52 ± 1.07	0.27 ± 0.02^b^
7	8	15.59 ± 1.30	−13.63 ± 8.09^ab^	0.98 ± 0.02	0.79 ± 0.03	12.27 ± 0.90	12.08 ± 1.05	0.24 ± 0.02^ab^
14	8	15.50 ± 1.27	−17.84 ± 8.06^a^	0.97 ± 0.06	0.80 ± 0.02	11.92 ± 1.44	11.20 ± 1.07	0.25 ± 0.05^a^
21	8	15.46 ± 1.24	−21.45 ± 8.01^a^	0.96 ± 0.01	0.79 ± 0.03	12.47 ± 1.13	11.99 ± 1.04	0.21 ± 0.05^a^
Source	D.F							
Yogurt samples	3	NS	NS	NS	NS	NS	NS	NS
Storage time	3	NS	*	NS	NS	NS	NS	*
Error	24							
Total	31							

C0, control yogurt (0%); C1, yogurt with 1% coriander; C2, yogurt with 2% coriander; C3, yogurt with 3% coriander. ^a,b^means in the same column without a common superscript differ (p < 0.05). *significant at 0.05 probability levels. NS, not significant.

Sensory analysis provides information about on consumers perceive the properties of a product, which is crucial for product development. There is a wealth of empirical research demonstrating the important influence of the senses on our perceptions, our emotional responses to food, and our food choices (39–41). This influence of the senses can, of course, be influenced in many different ways, either independently or synergistically (42). In general, consumers’ expectations for quality, tasty and healthy products continue to increase. A consumer who identifies as someone who cares about nutrition and health is likely to buy functional foods. However, the taste and smell of food always play an important role in consumer acceptance (7). Therefore, there is always a risk of rejection of products with poor sensory quality.

**Figure 1:**
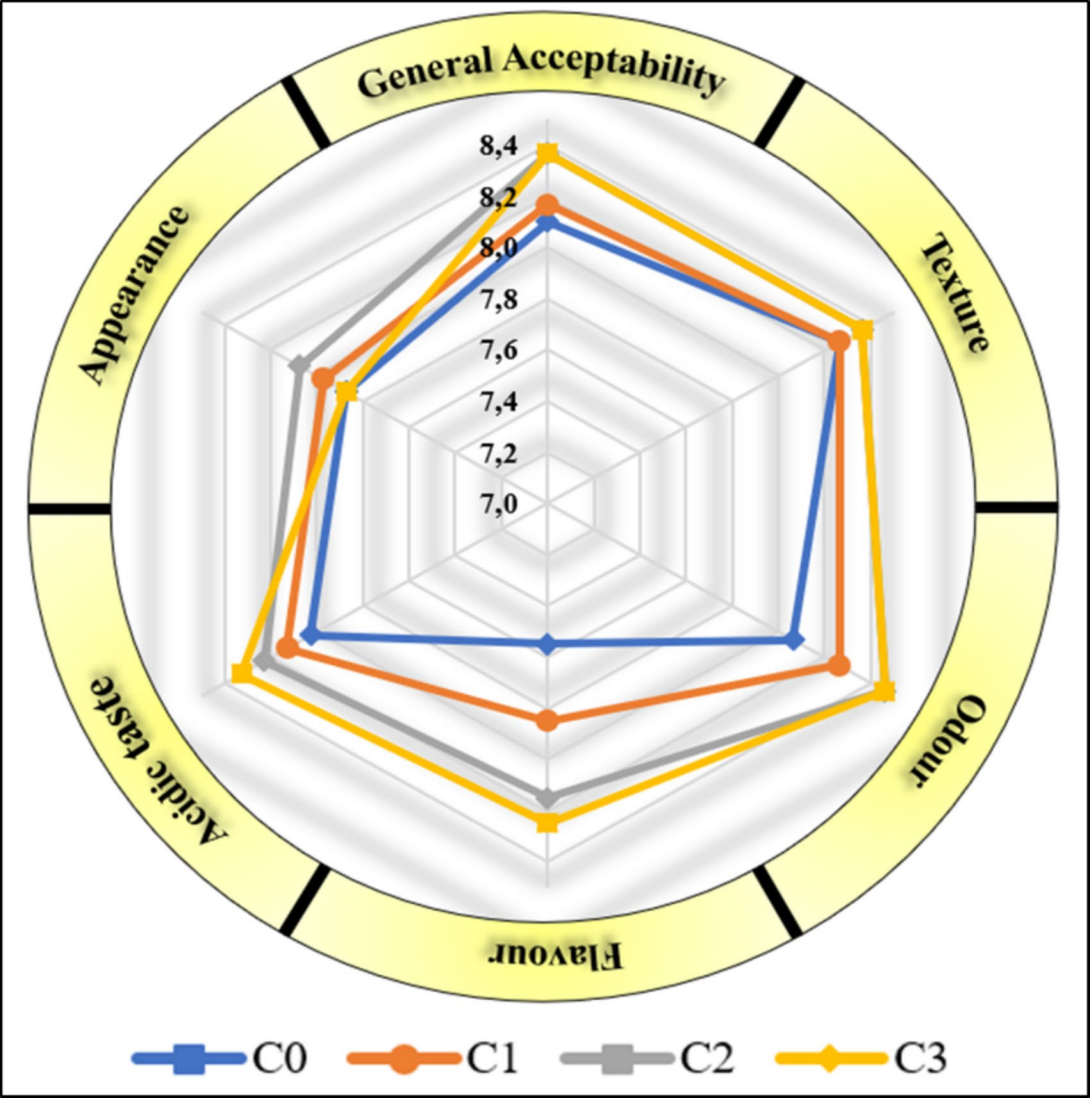
Sensory scores of the yogurt samples.

The sensory analysis results of the yogurt samples are summarized in Figure 1. The addition of coriander improved several sensory parameters such as appearance, odor, and texture of the yogurts. However, these changes did not result in significant differences in the mean values of these parameters (*p* > 0.05). C2 yogurts received highest score for appearance. The mean odor scores of all coriander yogurt samples was found to be higher than those of the C0 yogurt. The addition of coriander significantly influenced the taste and overall acceptability of yogurts (*p* < 0.05). However, it was found that the TA value, one of the physicochemical properties related to the taste of yogurt, was lower in CO yogurt. The yogurts with coriander received higher scores for flavor and acceptability than the C0 yogurt. The highest scores for flavor, odor, texture and acceptability were observed for the C2 and C3 yogurts (Figure 1). The addition of 3% coriander had a negative effect on the evaluation of appearance. The sensory scores of the yogurt samples exhibited a general decrease over the course of storage period. The acidic taste and odor gradually decreased with increasing storage time. This decrease is consistent with the pH value determined. This result can be attributed to the continued activity of the yogurt bacteria during the storage period.

There were significant differences in the overall acceptability of the storage times for yogurts. Panelists favored the general acceptability of the first day over the other days. Day 21 results were the least popular storage period due to the difference in aftertaste and high acidity.

## 4 Conclusion

This study was conducted with the addition of coriander to yogurt to determine whether the various parameters measured during the storage period developed in the same direction as in the control yogurts. The addition of coriander to yogurt resulted in a functional food that meets the expectations of health-conscious people. We investigated whether the various parameters measured during storage would develop in the same direction as the control yogurt. It was found that the addition of coriander had a significant effect on the TA, pH, WHC and syneresis values of the yogurt. The changes in TA and pH values were consistent with the syneresis value. The use of different proportions of coriander also significantly influenced the values of the color parameters. The color changes were relatively stable during storage. The L* and a* values decreased, but significant differences were only found for the a* value of the yogurts.

The number of *S. thermophilus* and *L. bulgaricus* was found to be lower in yogurt containing coriander than the C0 yogurt. The influence of coriander was found to have a significant effect only on the number of *S. thermophilus*. The results indicate that the use of aromatic medicinal plants can extend the shelf life of yogurt. The sensory results show that the addition of coriander makes a difference in terms of taste and overall acceptability. These samples achieved more acceptable scores. The panelists agreed that coriander can be added to yogurt at a level of 2%. In future studies, we may recommend investigating the potential of incorporating other medicinal aromatic plants (basil, bay leaf, dill) such as coriander into yogurt production.

## Data Availability

The original contributions presented in the study are included in the article/supplementary material, further inquiries can be directed to the corresponding author.
